# Longitudinal changes in pulmonary function and patient-reported outcomes after lung cancer surgery

**DOI:** 10.1186/s12931-022-02149-9

**Published:** 2022-08-30

**Authors:** Sumin Shin, Sunga Kong, Danbee Kang, Genehee Lee, Jong Ho Cho, Young Mog Shim, Juhee Cho, Hong Kwan Kim, Hye Yun Park

**Affiliations:** 1 Department of Thoracic and Cardiovascular Surgery, School of Medicine, Ewha Womans University, Seoul, South Korea; 2 Department of Thoracic and Cardiovascular Surgery, Samsung Medical Center, Sungkyunkwan University School of Medicine, 81 Irwon-Ro, Gangnam-Gu, Seoul, 06351 Korea; 3 Department of Clinical Research Design and Evaluation, SAIHST, Sungkyunkwan University, Seoul, South Korea; 4 Patient-Centered Outcomes Research Institute, Samsung Medical Center, Seoul, South Korea; 5 Center for Clinical Epidemiology, Samsung Medical Center, Seoul, South Korea; 6 Department of Epidemiology, Johns Hopkins University Bloomberg School of Public Health, Baltimore, MD USA; 7 Division of Pulmonary and Critical Care Medicine, Department of Medicine, Samsung Medical Center, Sungkyunkwan University, School of Medicine, 81 Irwon-Ro, Gangnam-Gu, Seoul, 06351 South Korea

**Keywords:** Non-small cell lung cancer, Patients reported outcomes, Pulmonary function, Surgery

## Abstract

**Background:**

Surgery is the mainstay of treatment for non-small cell lung cancer, but the decline in pulmonary function after surgery is noticeable and requires attention. This study aimed to evaluate longitudinal changes in pulmonary function and integrated patient-reported outcomes (PROs) after lung cancer surgery.

**Methods:**

Data were obtained from a prospective cohort study, the Coordinate Approach to Cancer Patients’ Health for Lung Cancer. Changes in forced vital capacity (FVC) and forced expiratory volume in 1 s (FEV_1_) at 2 weeks, 6 months, and 1 year after surgery, and the corresponding modified Medical Research Council (mMRC) dyspnea scale and chronic obstructive lung disease assessment test (CAT) scores were evaluated. Mixed effects model was used to investigate changes in pulmonary function and PROs.

**Results:**

Among 620 patients, 477 (76.9%) underwent lobectomy, whereas 120 (19.4%) and 23 (3.7%) were treated with wedge resection/segmentectomy and bilobectomy/pneumonectomy, respectively. Both FVC and FEV_1_ markedly decreased 2 weeks after surgery and improved thereafter; however, they did not recover to baseline values. The corresponding mMRC dyspnea scale and CAT scores worsened immediately after surgery. The dyspnea scale of the mMRC was still higher, while CAT scores returned to baseline one year after surgery, although breathlessness and lack of energy persisted. Compared to the changes from baseline of FVC and FEV_1_ in patients who underwent lobectomy, patients who underwent bilobectomy/pneumonectomy showed a greater decrease in FVC and FEV_1_, while wedge resection/segmentectomy patients had smaller decreases in FVC and FEV_1_ at 2 weeks, 6 months, and 1 year after surgery. Bilobectomy/pneumonectomy patients had the highest mMRC dyspnea grade among the three groups, but the difference was not statistically significant one year after surgery.

**Conclusions:**

After lung cancer surgery, pulmonary function and PROs noticeably decreased in the immediate post-operative period and improved thereafter, except for dyspnea and lack of energy. Proper information on the timeline of changes in lung function and symptoms following lung cancer surgery could guide patient care approaches after surgery.

*Trial registration:* ClinicalTrials.gov; No.: NCT03705546; URL: www.clinicaltrials.gov

**Supplementary Information:**

The online version contains supplementary material available at 10.1186/s12931-022-02149-9.

## Background

Lung cancer is the leading cause of cancer-related deaths in both men and women [1]. The median age at diagnosis of lung cancer is 70 years [2], and more than 70% of future lung cancer cases are expected to occur in adults older than 65 years [3]. Surgery is the best treatment option for patients with early stage non-small cell lung cancer (NSCLC) [4], and the 5-year survival rate among patients with stage I NSCLC after curative resection has increased to 70% [5]. Despite this improvement in survival rate, a decrease in pulmonary function after surgery is inevitable. Accordingly, several studies have shown serial changes in pulmonary function after lung resection [6–9]. Lung function dropped sharply until 1 month, partly recovered at 3 months, and stabilized at 6 months after surgery [6–9]. These changes in lung function vary depending on the extent of surgery, with the reduction in forced expiratory volume in 1 s (FEV_1_) being 9% and 35% after lobectomy and pneumonectomy, respectively [7, 8].

Lung cancer survivors commonly suffer from post-treatment symptoms, such as pain, dyspnea, and fatigue, which negatively affect their quality of life (QOL) [10–14]. Given the importance of health-related quality of life (HRQOL) in patients with lung cancer and its prognostic impact, assessment of patient-reported outcomes (PROs) has been emphasized [15]. In particular, lung cancer-specific HRQOL domains include dyspnea and cough [16, 17] influencing in a sedentary lifestyle and physical function ability [18, 19]. In addition, several studies have found that postoperative respiratory symptoms are more frequently observed in patients with low pulmonary function; however, the changes in PROs were measured between baseline and only one point after surgical resection. There are limited data on the changes in PROs that are associated with changes in pulmonary function over time after surgical resection during longitudinal follow-up. Thus, we conducted a longitudinal cohort study to examine serial changes in pulmonary function and the associated changes in PRO in NSCLC patients undergoing curative resection.

## Methods

**Fig. 1:**
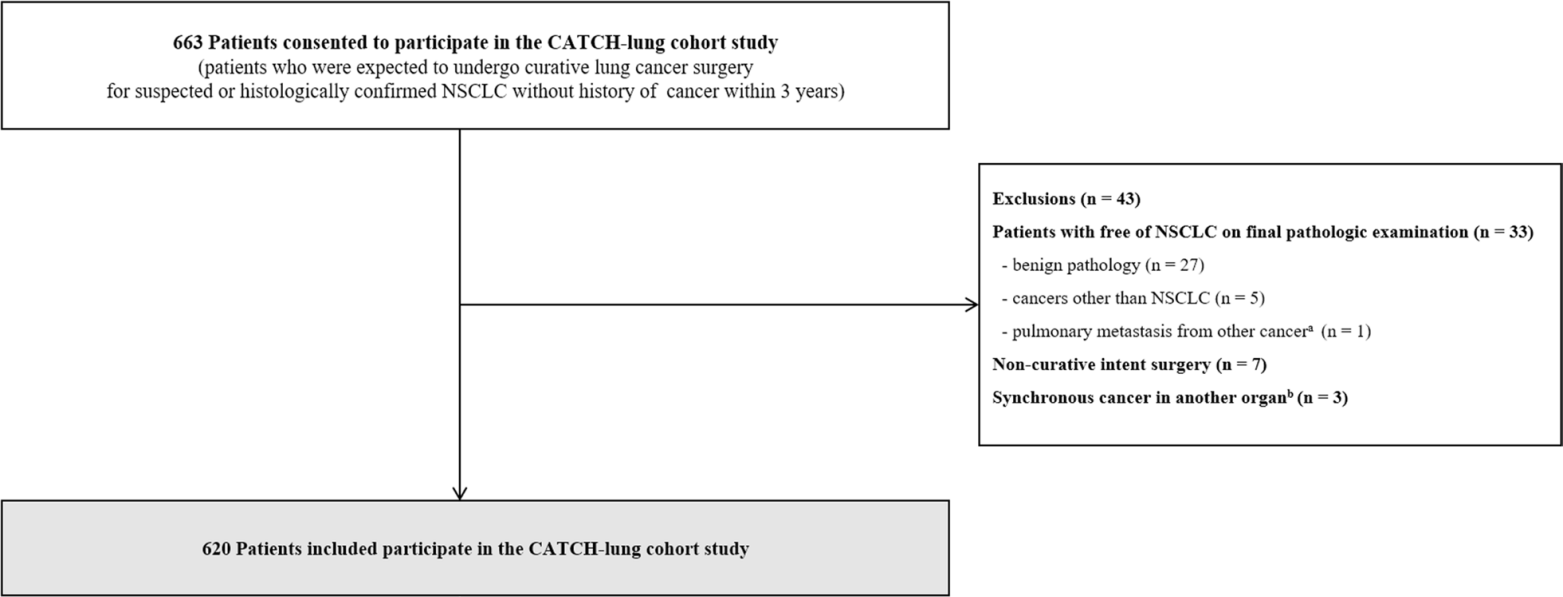
Flow chart of study participants. a. pulmonary metastasis of cancer in other organs. b. patients who were simultaneously diagnosed with cancer in other organs. *CATCH –LUNG* Coordinate Approach to Cancer Patient’s Health for Lung Cancer, *NSCLC* non-small cell lung cancer

We used data from a prospective cohort study called the Coordinated Approach to Cancer Patients’ Health for Lung Cancer (CATCH-LUNG), which recruited patients expected to undergo curative lung cancer surgery for suspected NSCLC at Samsung Medical Center in Seoul, Korea from March 2016 to October 2018. The patient inclusion criteria were as follows: (1) expected to undergo curative lung cancer surgery for suspected or histologically confirmed NSCLC, (2) Eastern Cooperative Oncology Group performance status of either 0 or 1, (3) no problems in walking, and (4) understood the purpose of the study and agreed to participate in it. Patients were excluded if they were free of NSCLC on final pathologic examination and if they had either benign pathology (n = 27), cancers other than NSCLC (n = 5), or pulmonary metastasis from other cancers (n = 1). In addition, patients who were diagnosed with disseminated lung cancer in the operative field thus did not undergo curative intent surgery were excluded (n = 7), as were patients who had synchronous cancer in another organ (n = 3). Finally, among 663 patients, 620 were included in the analysis (Fig. 1). The protocols for patient enrollment and data collection are described in a previous study [20]. The study protocol was approved by the Institutional Review Board of Samsung Medical Center (no. 2015–11-025), and written informed consent was obtained from all participants.

### Measurements

Pulmonary function and PROs were measured before surgery and repeated at 2 weeks (median, 2 weeks), 6 months (median, 5 months), and 1 year (median, 11.2 months) after surgery. Pulmonary function measurements, including spirometry and diffusing capacity of the lung for carbon monoxide (DLco), were performed using a Vmax 22 respiratory analyzer (SensorMedics, OH, USA) according to the American Thoracic Society/European Respiratory Society criteria [21, 22]. Absolute values of FEV_1_, forced vital capacity (FVC), and DLco were obtained, and their percent of the predicted values (% pred) were calculated using a representative Korean sample [23, 24] as a reference. An obstructive spirometric pattern was defined as FEV_1_/FVC < 70%, and a restrictive spirometric pattern was defined as both FEV_1_/FVC ≥ 70% and FVC < 80% pred. PROs were assessed using the modified Medical Research Council (mMRC) dyspnea scale and chronic obstructive lung disease assessment test (CAT) score. The mMRC dyspnea scale is a questionnaire consisting of five statements related to perceived breathlessness, which are classified into grades 0 to 4 [25]. The CAT score consists of eight parameters: cough, sputum, chest tightness, dyspnea, activity confidence, sleep, and energy. The scores range from 0 to 5 points, resulting in a total CAT score ranging from 0 to 40 points [26].

Sociodemographic and behavioral information of the participants, including age, sex, body mass index, smoking status, and comorbidities, were obtained from electronic medical records. Clinical information, including cell type, surgery type, video-assisted thoracic surgery, postoperative pulmonary complications (PPC), and adjuvant treatmen were also collected after surgery. PPC were defined as any of the following conditions: (1) atelectasis requiring bronchoscopic toileting, (2) pneumonia (at least three of leukocytosis, pulmonary infiltrate or consolidation, fever [> 38 °C], culture-positive, or use of antibiotics), (3) acute lung injury or acute respiratory distress syndrome (rate of arterial oxygen partial pressure to fractional inspired oxygen < 300 and bilateral infiltrate observed on chest radiograph without evidence of congestive heart failure or volume overload), or (4) acute exacerbation of chronic obstructive pulmonary disease [27].

### Statistical analysis

We used mixed effects model for longitudinal data analysis and modeled changes in absolute values of FVC, FEV_1_ and DLco; % pred for FVC, FEV_1_, and DLco; FEV_1_/FVC; and prevalence of obstructive or restrictive spirometric patterns at each time point. These mixed effects model provided the average longitudinal change from preoperative values (with 95% confidence intervals [CI]) and allowed for random variations in longitudinal changes among participants according to normal distributions with unstructured variance–covariance matrices (See Additional file 1 containing information regarding the formula of the mixed effects model). Generalized estimating equation with binomial as the family and logit as the link function was used to calculate prevalence ratio for FEV_1_/FVC, obstructive pattern and restrictive pattern comparing to the baseline. Adjusted mean and proportion were obtained from the models. Furthermore, we compared changes from baseline of pulmonary function at each time point according to the type of surgery (bilobectomy/pneumonectomy, lobectomy, and wedge resection/segmentectomy). We performed sensitivity analysis in patients with normal lung function before surgery.

In terms of PROs, patients grade 2 scores or higher on the mMRC dyspnea scale (I get short of breath when hurrying on a level or up a slight hill) were considered to have significant dyspnea in the current study [25]. Based on CAT total scores, patients were categorized into three groups, low (0 ≤ CAT < 10), medium (10 ≤ CAT < 20), and high (20 ≤ CAT ≤ 40) impact groups, according to the CAT user guide (http://www.catestonline.org). To evaluate the change in the prevalence of mMRC ≥ 2 and medium or high CAT (total score ≥ 10), we used a generalized estimating equation with binomial as the family and logit as the link function. To adjust for confounding factors, we included age, sex, stage, obesity, smoking status, cell type, surgery type, video-assisted thoracic surgery, postoperative pulmonary complications, and adjuvant treatment.

All reported *P-*values were set at a significance level of 0.05. Statistical analyses were performed using Stata version 16 (StataCorp LLC, College Station, TX).

## Results

### Patient characteristics

The mean (standard deviation, SD) age of study participants was 61.2 (9.0) years, and the percentage of male patients was 56.5% (n = 350) (Table 1). Among the 620 eligible participants, 23 (3.7%), 477 (79.9%), and 120 (19.4%) underwent bilobectomy/pneumonectomy, lobectomy, and wedge resection/segmentectomy, respectively. All the participants completed the baseline examination, and 603 (97.3%, bilobectomy/pneumonectomy n = 21; lobectomy n = 464; wedge resection/segmentectomy n = 118), 536 (86.6%, bilobectomy/pneumonectomy n = 20; lobectomy n = 406; wedge resection /segmentectomy n = 110), and 518 (83.5%, bilobectomy/pneumonectomy n = 17; lobectomy n = 395; wedge resection/segmentectomy n = 106) completed the examinations at 2 weeks, 6 months, and 1 year after surgery, respectively. Compared to patients who underwent lobectomy, bilobectomy/pneumonectomy was more associated with parameters such as old age, male sex, ever smoker, squamous cell carcinoma, advanced stage, and adjuvant treatment (Table 1).

**Table 1:** Clinical and surgical characteristics of the study participants

		Type of surgery			*P*-value
	Overall (N = 620)	Wedge resection/segmentectomy	Lobectomy	Bilobectomy/pneumonectomy	
Characteristic		(n = 120, 19.4%)	(n = 477, 76.9%)	(n = 23, 3.7%)	
Age, years	61.2 (9.0)	59.6 (9.2)	61.5 (9.0)	63.2 (7.8)	0.07
Sex, male	350 (56.5)	63 (52.5)	268 (56.2)	19 (82.6)	0.03
Body mass index, kg/m^2^	24.0 ± 2.9				
Smoking status, ever smoker	319 (51.5)	54 (45.0)	247 (51.8)	18 (78.3)	0.01
Comorbidities					
COPD^a^	140 (22.8)	26 (21.7)	106 (22.2)	8 (34.8)	0.22
Hypertension	211 (34.0)	31 (25.8)	170 (35.6)	10 (43.5)	0.08
Diabetes mellitus	92 (14.8)	14 (11.7)	73 (15.3)	5 (21.7)	0.39
Cardiovascular disease	16 (2.6)	4 (3.3)	12 (2.5)	0	0.64
Cell type					< 0.01
Adenocarcinoma	513 (82.7)	111 (92.5)	394 (82.6)	8 (34.8)	
Squamous cell	87 (14.0)	6 (5.0)	67 (14.1)	14 (60.9)	
Others	20 (3.2)	3 (2.5)	16 (3.4)	1 (4.4)	
Tumor size, cm	2.7 (1.5)	1.7 (0.7)	2.9 (1.5)	4.1 (1.9)	< 0.01
Pathologic stage					< 0.01
I	451 (72.7)	116 (96.7)	330 (69.2)	5 (21.7)	
II	96 (15.5)	2 (1.7)	83 (17.4)	11 (47.8)	
III	70 (3.0)	2 (1.7)	62 (13.0)	6 (26.1)	
IV	3 (3.2)	0	2 (0.4)	1 (4.4)	
Surgical approach (VATS, %)	490 (79.0)	112 (93.3)	373 (78.2)	5 (21.7)	< 0.01
PPC, yes	49 (7.9)	10 (8.3)	36 (7.6)	3 (13.0)	0.62
Adjuvant treatment, yes	149 (24.0)	3 (2.5)	131 (27.5)	15 (65.2)	< 0.01

Values in the table are presented as mean ± standard deviation or n (%)

*COPD* chronic obstructive pulmonary disease, *VATS* video-assisted thoracic surgery, *PPC* postoperative pulmonary complications

^a^COPD is defined as a pre-bronchodilator forced expiratory volume in 1 s/forced expiratory vital capacity < 70%

### Changes in pulmonary function

**Fig. 2:**
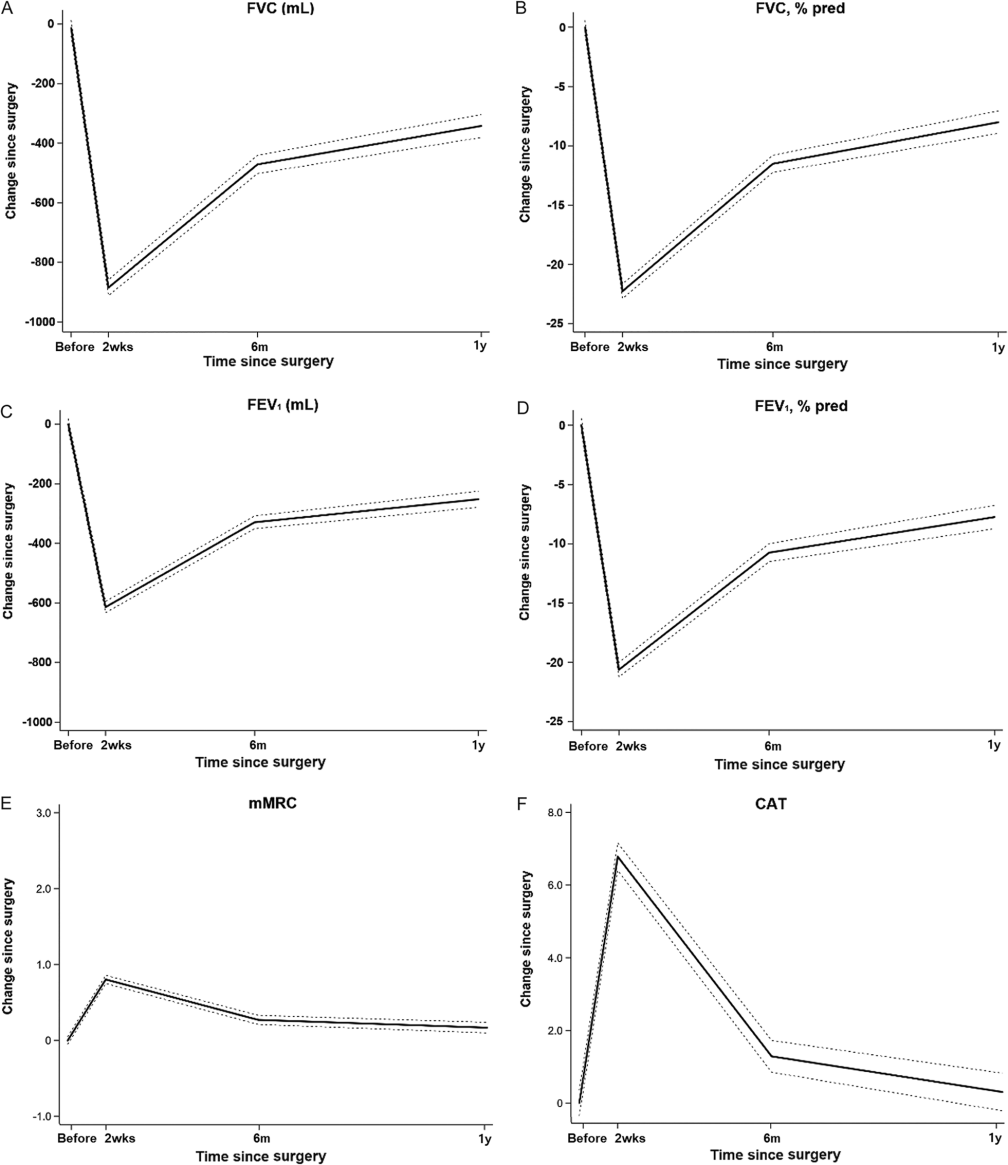
Change in pulmonary function and patient-reported outcomes by postoperative time. (**A**) FVC (mL), (**B**) FVC (percent of the predicted value), (**C**) FEV_1 _(mL), (**D**) FEV_1_ (percent of the predicted value) (**E**), mMRC dyspnea scale, (**F**) CAT *CAT* chronic obstructive pulmonary disease assessment test, *FEV*_*1*_ forced expiratory volume in 1 s, *FVC* forced expiratory vital capacity, *mMRC dyspnea scale* modified Medical Research Council dyspnea scale

The baseline, average (SD) levels were 3595.1 mL and 92.9% pred for FVC, 2637.4 mL and 90.1% pred for FEV_1_, and 18.4 ml/min/mmHg and 89.9% pred for DLco. The adjusted means were 3600.3 mL and 93.2% pred for FVC, 2647.0 mL and 90.4% pred for FEV_1_, and 18.6 ml/min/mmHg and 90.5% pred for DLco (Table 2). FVC decreased sharply 2 weeks after surgery; it increased thereafter, but it did not return to baseline levels (2721.4 mL, 3137.7 mL, and 3268.8 mL at 2 weeks, 6 months, and 1 year after surgery, respectively) (Table 2). Compared to baseline, patients showed reduced FVC of 331.6 mL at 1 year after surgery (Fig. 2). Furthermore, compared to baseline, patients showed declines in % pred of FVC (95% CI) over the follow-up period: -23.3% (-24.1, -22.5), -12.3% (-13.2, -11.4), and -8.5% (-9.4, -7.6) at 2 weeks, 6 months, and 1 year after surgery, respectively (Fig. 2). A similar pattern was observed for FEV_1_ (mL and % pred) and DLCO (ml/min/mmHg and % pred) (Table 2, Fig. 2). During follow-up, compared to baseline, the prevalence of restrictive patterns increased by 7.8, 4.5, and 3.2 times at 2 weeks, 6 months, and 1 year after surgery, respectively, whereas the prevalence of obstructive patterns was similar (Table 3). In sensitivity analyses of participants with normal lung function at baseline (n = 431. 69.5%), 10.4%, 12.0%, and 12.3% of patients had incidents of the obstructive pattern at 2 weeks, 6 months, and 1 year after surgery, respectively. On the other hand, 61.7%, 29.8%, and 20.4% of patients had incidents of the restrictive pattern at 2 weeks, 6 months, and 1 year after surgery, respectively (See Additional file 2).

**Table 2:** Changes in pulmonary function from baseline to 2 weeks, 6 months, and 1 year after surgery

			Before surgery	2 weeks after surgery	6 months after surgery	1 year after surgery
Overall						
FVC (mL)						
Adjusted mean (SE)			3600.3 (21.2)	2721.4 (21.5)	3137.7 (23.1)	3268.8 (24.8)
Change from baseline (95% CI)^a^			Reference	− 879 (− 910.3, − 847.6)	− 462.6 (− 495.4, − 429.8)	− 331.6 (− 365.4, − 297.7)
FVC, % pred						
Adjusted mean (SE)			93.2 (0.5)	70.9 (0.5)	81.6 (0.5)	85.2 (0.6)
Change from baseline (95% CI)^a^			Reference	− 23.3 (− 24.1, − 22.5)	− 12.3 (− 13.2, − 11.4)	− 8.5 (− 9.4, − 7.6)
FEV_1_ (mL)						
Adjusted mean (SE)			2647.0 (16.7)	2033.1 (16.9)	2318.3 (17.9)	2395.2 (18.9)
Change from baseline (95% CI)^a^			Reference	− 613.9 (− 636.2, − 591.6)	− 328.7 (− 352.0, − 305.3)	− 251.7 (− 275.7, − 227.8)
FEV_1_, % pred						
Adjusted mean (SE)			90.4 (0.5)	69.8 (0.5)	79.6 (0.6)	82.6 (0.6)
Change from baseline (95% CI)^a^			Reference	− 20.6 (− 21.3, − 19.9)	− 10.7 (− 11.5, − 10.0)	− 7.8 (− 8.6, − 7.0)
DLco, ml/min/mmHg						
Adjusted mean (SE)			18.6 (0.1)		15.8 (0.1)	15.9 (0.1)
Change from baseline (95% CI)^a^			Reference		− 4.0 (− 5.1, − 2.9)	− 3.3 (− 4.4, − 2.2)
DLco, % pred						
Adjusted mean (SE)			90.5 (0.6)		77.5 (0.6)	78.3 (0.6)
Change from baseline (95% CI)^a^			Reference		− 13.0 (− 14.0, − 12.0)	− 12.2 (− 13.2, − 11.2)
Type of surgery						
FVC (mL)						
Adjusted mean (SE)						
Wedge resection/segmentectomy			3648.0 (49.2)	2977.9 (50)	3403.6 (52.8)	3483.3 (56.4)
Lobectomy			3596.2 (24.2)	2678.7 (24.6)	3102.5 (26.3)	3242.0 (28.1)
Bilobectomy/pneumonectomy			3394.4 (116.5)	2198.8 (119.8)	2394.8 (125.6)	2624.3 (136.5)
* P-*values			0.15	< 0.01	< 0.01	< 0.01
Change from baseline (95% CI)^a^						
Wedge resection/segmentectomy			Reference	− 670.1 (− 739, − 601.2)	− 244.4 (− 315.4, − 173.4)	− 164.7 (− 237.9, − 91.5)
Lobectomy			Reference	− 917.4 (− 952.1, − 882.7)	− 493.6 (− 530.2, − 457.0)	− 354.2 (− 391.8, − 316.6)
Bilobectomy/pneumonectomy			Reference	− 1195.6 (− 1358.3, 1032.9)	− 999.6 (− 1165.9, − 833.3)	− 770.1 (− 949.4, − 590.8)
* P* for interaction				< 0.01	< 0.01	< 0.01
FVC, % predicted						
Adjusted mean (SE)						
Wedge resection/segmentectomy			93.4 (1.1)	76.4 (1.1)	87.1 (1.2)	89.3 (1.3)
Lobectomy			93.3 (0.5)	70.0 (0.5)	81.0 (0.6)	84.7 (0.6)
Bilobectomy/pneumonectomy			88.9 (2.6)	60.2 (2.7)	65.6 (2.8)	70.8 (3.1)
* P-*values			0.25	< 0.01	< 0.01	< 0.01
Change from baseline (95% CI)^a^						
Wedge resection/segmentectomy			Reference	− 17.0 (− 18.6, − 15.4)	− 6.4 (− 8.1, − 4.7)	− 4.1 (− 5.9, − 2.3)
Lobectomy			Reference	− 23.3 (− 24.1, − 22.5)	− 12.3 (− 13.2, − 11.4)	− 8.5 (− 9.4, − 7.6)
Bilobectomy/pneumonectomy			Reference	− 28.8 (− 32.6, − 24.9)	− 23.3 (− 27.3, − 19.3)	− 18.2 (− 22.5, − 13.9)
* P* for interaction				< 0.01	< 0.01	< 0.01
FEV_1_ (mL)						
Adjusted mean (SE)						
Wedge resection/segmentectomy			2677.9 (38.9)	2206.5 (39.4)	2485.5 (41.1)	2537.4 (43.4)
Lobectomy			2647.2 (19.1)	2004.8 (19.3)	2295.1 (20.4)	2376.9 (21.6)
Bilobectomy/pneumonectomy			2449.9 (92.1)	1669.6 (94.3)	1872.2 (97.9)	1981.1 (104.7)
* P-*values			0.08	< 0.01	< 0.01	< 0.01
Change from baseline (95% CI)^a^						
Wedge resection/segmentectomy			Reference	− 471.3 (− 520.9, − 421.8)	− 192.4 (− 243.4, − 141.4)	− 140.5 (− 192.8, − 88.2)
Lobectomy			Reference	− 642.4 (− 667.4, − 617.5)	− 352.1 (− 378.4, − 325.8)	− 270.3 (− 297.2, − 243.5)
Bilobectomy/pneumonectomy			Reference	− 780.3 (− 897.4, − 663.3)	− 577.7 (− 697.2, − 458.2)	− 468.8 (− 596.9, − 340.7)
* P* for interaction				< 0.01	< 0.01	< 0.01
FEV_1_, % pred						
Adjusted mean (SE)						
Wedge resection/segmentectomy			90.3 (1.2)	74.8 (1.2)	84.2 (1.3)	86.3 (1.3)
Lobectomy			90.6 (0.6)	68.9 (0.6)	79.0 (0.6)	82.2 (0.7)
Bilobectomy/pneumonectomy			85.2 (2.9)	61.3 (3)	66.8 (3)	70.8 (3.2)
* P*-values			0.19	< 0.01	< 0.01	< 0.01
Change from baseline (95% CI)^a^						
Wedge resection/segmentectomy			Reference	− 15.5 (− 17.1, − 13.8)	− 6.0 (− 7.7, − 4.3)	− 4.0 (− 5.8, − 2.1)
Lobectomy			Reference	− 21.7 (− 22.6, − 20.9)	− 11.6 (− 12.5, − 10.7)	− 8.4 (− 9.4, − 7.5)
Bilobectomy/pneumonectomy			Reference	− 24.0 (− 27.8, − 20.1)	− 18.4 (− 22.4, − 14.5)	− 14.4 (− 18.9, − 10)
* P* for interaction				< 0.01	< 0.01	< 0.01
DLco, ml/min/mmHg						
Adjusted mean (SE)						
Wedge resection/segmentectomy			18.4 (0.3)		16.9 (0.3)	16.7 (0.3)
Lobectomy			18.6 (0.1)		15.6 (0.2)	15.7 (0.1)
Bilobectomy/pneumonectomy			18.4 (0.7)		14.4 (0.8)	15.0 (0.7)
* P-*values			0.81		< 0.01	< 0.01
Change from baseline (95% CI)^a^						
Wedge resection/segmentectomy			Reference		− 1.5 (− 1.9, − 1.1)	− 1.7 (− 2.2, − 1.3)
Lobectomy			Reference		− 3.0 (− 3.2, − 2.8)	− 2.9 (− 3.1, − 2.6)
Bilobectomy/pneumonectomy			Reference		− 4.0 (− 5.1, − 2.9)	− 3.3 (− 4.4, − 2.2)
* P*-values					< 0.01	< 0.01
DLco, % pred						
Adjusted mean (SE)						
Wedge resection/segmentectomy			88.4 (1.3)		81.2 (1.3)	81.1 (1.4)
Lobectomy			91.0 (0.6)		76.6 (0.7)	77.6 (0.7)
Bilobectomy/pneumonectomy			90.1 (3.1)		71.7 (3.4)	75.4 (3.3)
* P-*values			0.21		< 0.01	0.05
Change from baseline (95% CI)^a^						
Wedge resection/segmentectomy			Reference		− 7.1 (− 0.3, − 5.1)	− 7.3 (− 9.5, − 5.1)
Lobectomy			Reference		− 14.4 (− 15.5, − 13.3)	− 13.4 (− 14.5, − 12.3)
Bilobectomy/pneumonectomy			Reference		− 18.5 (− 23.8, − 13.1)	− 14.8 (− 20.2, − 9.4)
* P*-values					< 0.01	< 0.01

^a^Adjusted for age, sex, stage, obesity, smoking status, cell type, type of surgery, video-assisted thoracic surgery, postoperative pulmonary complications, and adjuvant treatment

CI, confidence interval; DLco, diffusing lung capacity of the lung for carbon monoxide; FEV_1_, forced expiratory volume in 1 s; FVC, forced expiratory vital capacity; SE, standard error; % pred (percent of the predicted value)

**Table 3:** Changes in FEV_1_/FVC and patterns of ventilatory defect from baseline to 2 weeks, 6 months, and 1 year after surgery

			Before surgery	2 weeks after surgery	6 months after surgery	1 year after surgery
Overall						
FEV_1_/FVC (%)						
Adjusted mean (SE)			73.9 (0.3)	75.1 (0.3)	74.2 (0.3)	73.6 (0.4)
Change from baseline (95% CI)^a^			Reference	1.3 (0.8, 1.7)	0.4 (− 0.1, 0.9)	− 0.3 (− 0.8, 0.3)
Obstructive pattern						
Adjusted proportion (SE)			20.2 (1.5)	20.4 (1.6)	22.1 (1.6)	25.8 (1.8)
Odds ratio (95% CI)^a^			Reference	1.0 (0.8, 1.1)	1.0 (0.8, 1.2)	1.2 (1.0, 1.3)
Restrictive pattern						
Adjusted proportion (SE)			7.4 (1.0)	57.8 (1.9)	33.1 (1.9)	23.5 (1.8)
Odds ratio (95% CI)^a^			Reference	7.8 (6.0, 10.3)	4.5 (3.4, 5.8)	3.2 (2.4, 4.1)
Type of surgery						
FEV_1_/FVC (%)						
Adjusted mean (SE)						
Wedge resection/segmentectomy			73.6 (0.7)	74.7 (0.7)	73.4 (0.8)	73.1 (0.8)
Lobectomy			74.0 (0.3)	75.3 (0.4)	74.3 (0.4)	73.6 (0.4)
Pneumonectomy/bilobectomy			73.0 (1.7)	74.6 (1.7)	77.4 (1.8)	75.5 (2.0)
* P*-values			0.79	0.73	0.12	0.55
Obstructive pattern						
Adjusted proportion (SE)						
Wedge resection/segmentectomy			25.1 (3.8)	22.4 (3.7)	24.4 (3.9)	27.2 (4)
Lobectomy			21.7 (1.7)	21.7 (1.7)	22.3 (1.8)	25.8 (1.9)
Bilobectomy/pneumonectomy			23.0 (7.3)	17.3 (6.7)	13.5 (6.1)	22.0 (7.7)
* P*-values			0.69	0.83	0.44	0.85
Restrictive pattern						
Adjusted proportion (SE)						
Wedge resection/segmentectomy			4.9 (2)	44.6 (4.5)	15.5 (3.4)	9.1 (2.8)
Lobectomy			6.8 (1.1)	60.0 (2.2)	35.8 (2.3)	25.7 (2.1)
Bilobectomy/pneumonectomy			26.7 (9)	72.3 (10.4)	67.0 (10.9)	48.2 (11.7)
* P*-values			< 0.01	< 0.01	< 0.01	< 0.01

An obstructive pattern was defined as FEV_1_/FVC < 70%; a restrictive pattern was defined as both FEV_1_/FVC ≥ 70% and FVC < 80% predicted

*CI* confidence interval, *FEV*_*1*_ forced expiratory volume in 1 s; *FVC* forced expiratory vital capacity, *SE* standard error

^a^Adjusted for age, sex, stage, obesity, smoking status, cell type, type of surgery, video-assisted thoracic surgery, postoperative pulmonary complications, and adjuvant treatment

### Changes of PROs

Both the mMRC dyspnea scale and CAT scores decreased at 2 weeks after surgery and were alleviated over time (Table 4, Fig. 2). The prevalence of subjects with mMRC ≥ 2 was increased to 15.2, 3.3, and 2.1 times that of baseline at 2 weeks, 6 months, and 1 year after surgery, respectively. The prevalence of subjects with CAT scores ≥ 10 also increased by 6.0 and 1.4 times that of baseline at 2 weeks and 6 months after surgery, respectively, although CAT scores fully recovered at 1 year (Table 5). The individual changes in CAT levels are listed in Table 5. Two weeks post-surgery, all domains except the amount of phlegm were significantly worse than at baseline; only the breathlessness walking upstairs and lack of energy domains of the CAT persisted 1 year after surgery.

**Table 4:** Changes in patient-reported outcomes from baseline to 2 weeks, 6 months, and 1 year after surgery

			Before surgery	2 weeks after surgery	6 months after surgery	1 year after surgery
Overall						
mMRC dyspnea scale						
Adjusted mean (SE)			0.25 (0.03)	1.05 (0.03)	0.53 (0.03)	0.42 (0.03)
Change from baseline (95% CI)^a^			Reference	0.81 (0.74, 0.88)	0.28 (0.21, 0.36)	0.17 (0.10, 0.24)
CAT total score						
Adjusted mean (SE)			5.97 (0.23)	12.77 (0.24)	7.29 (0.25)	6.22 (0.25)
Change from baseline (95% CI)^a^			Reference	6.79 (6.3, 7.29)	1.32 (0.8, 1.84)	0.24 (− 0.29, 0.77)
Type of surgery						
mMRC dyspnea scale						
Adjusted mean (SE)						
Wedge resection/segmentectomy			0.27 (0.06)	0.82 (0.06)	0.36 (0.07)	0.34 (0.07)
Lobectomy			0.24 (0.03)	1.09 (0.03)	0.55 (0.03)	0.42 (0.03)
Bilobectomy/pneumonectomy			0.13 (0.14)	1.44 (0.15)	0.83 (0.16)	0.65 (0.17)
* P*-values			0.67	< 0.01	< 0.01	0.22
Change from baseline (95% CI)^a^						
Wedge resection/segmentectomy			Reference	0.55 (0.39, 0.71)	0.10 (− 0.06, 0.26)	0.07 (− 0.09, 0.24)
Lobectomy			Reference	0.85 (0.76, 0.93)	0.30 (0.22, 0.39)	0.17 (0.09, 0.26)
Bilobectomy/pneumonectomy			Reference	1.31 (0.95, 1.67)	0.78 (0.41, 1.16)	0.52 (0.13, 0.91)
* P* for interaction				< 0.01	< 0.01	0.11
CAT total score						
Adjusted mean (SE)						
Wedge resection/segmentectomy			6.08 (0.54)	11.61 (0.55)	6.55 (0.55)	5.78 (0.54)
Lobectomy			5.83 (0.27)	13.09 (0.28)	7.42 (0.28)	6.28 (0.27)
Bilobectomy/pneumonectomy			8.48 (1.27)	12.55 (1.3)	8.75 (1.34)	6.96 (1.31)
* P*-values			0.12	0.02	0.12	0.2
Change from baseline^a^						
Wedge resection/segmentectomy			Reference	5.54 (4.43, 6.64)	0.47 (− 0.67, 1.61)	− 0.30 (− 1.48, 0.89)
Lobectomy			Reference	7.26 (6.69, 7.83)	1.59 (1.00, 2.18)	0.45 (− 0.15, 1.05)
Bilobectomy/pneumonectomy			Reference	4.07 (1.53, 6.61)	0.27 (-2.46, 3.00)	− 1.52 (− 4.31, 1.27)
* P* for interaction				< 0.01	0.17	0.25

*CAT* chronic obstructive pulmonary disease assessment test, *CI* confidence interval, *mMRC dyspnea scale* modified Medical Research Council dyspnea scale, *SE* standard error

^a^Adjusted for age, sex, stage, obesity, smoking status, cell type, type of surgery, video-assisted thoracic surgery, postoperative pulmonary complications, and adjuvant treatment

**Table 5:** Change in the COPD assessment test results from baseline to 2 weeks, 6 months, and 1 year after surgery

		Before surgery	2 weeks after surgery	6 months after surgery	1 year after surgery
Cough frequency					
Adjusted mean (SE)		0.82 (0.05)	2.00 (0.05)	0.96 (0.06)	0.68 (0.06)
Change from baseline (95% CI)^a^		Reference	1.18 (1.05, 1.31)	0.15 (0.01, 0.28)	− 0.14 (− 0.27, 0.00)
Amount of phlegm					
Adjusted mean (SE)		0.83 (0.05)	0.89 (0.05)	0.77 (0.05)	0.67 (0.05)
Change from baseline (95% CI)^a^		Reference	0.05 (− 0.06, 0.16)	− 0.06 (− 0.18, 0.05)	− 0.16 (− 0.27, − 0.04)
Chest tightness					
Adjusted mean (SE)		0.50 (0.05)	1.30 (0.05)	0.50 (0.05)	0.47 (0.05)
Change from baseline (95% CI)^a^		Reference	0.8 (0.68, 0.91)	− 0.01 (− 0.12, 0.11)	− 0.04 (− 0.15, 0.08)
Breathlessness walking upstairs					
Adjusted mean (SE)		1.28 (0.06)	2.85 (0.06)	1.97 (0.06)	1.79 (0.07)
Change from baseline (95% CI)^a^		Reference	1.57 (1.43, 1.71)	0.70 (0.55, 0.84)	0.52 (0.37, 0.66)
Home activities limited					
Adjusted mean (SE)		0.05 (0.03)	0.64 (0.03)	0.16 (0.04)	0.10 (0.04)
Change from baseline (95% CI)^a^		Reference	0.59 (0.51, 0.68)	0.12 (0.03, 0.21)	0.06 (− 0.04, 0.15)
Not confident leaving home					
Adjusted mean (SE)		0.08 (0.04)	1.26 (0.04)	0.23 (0.05)	0.15 (0.05)
Change from baseline (95% CI)^a^		Reference	1.19 (1.07, 1.3)	0.16 (0.04, 0.28)	0.08 (− 0.04, 0.2)
Sleep disturbance					
Adjusted mean (SE)		0.96 (0.06)	1.56 (0.07)	0.86 (0.07)	0.86 (0.07)
Change from baseline (95% CI)^a^		Reference	0.60 (0.44, 0.75)	− 0.10 (− 0.26, 0.06)	− 0.42 (− 0.58, − 0.25)
Lack of energy					
Adjusted mean (SE)		1.49 (0.06)	2.50 (0.06)	1.84 (0.06)	1.80 (0.06)
Change from baseline (95% CI)^a^		Reference	1.00 (0.87, 1.14)	0.35 (0.21, 0.49)	0.31 (0.17, 0.46)

*COPD* chronic obstructive pulmonary disease, *CI* confidence interval, *SE* standard error

^a^Adjusted for age, sex, stage, obesity, smoking status, cell type, type of surgery, video-assisted thoracic surgery, postoperative pulmonary complications, and adjuvant treatment

### Impact of surgical extent on pulmonary function and PROs

**Fig. 3:**
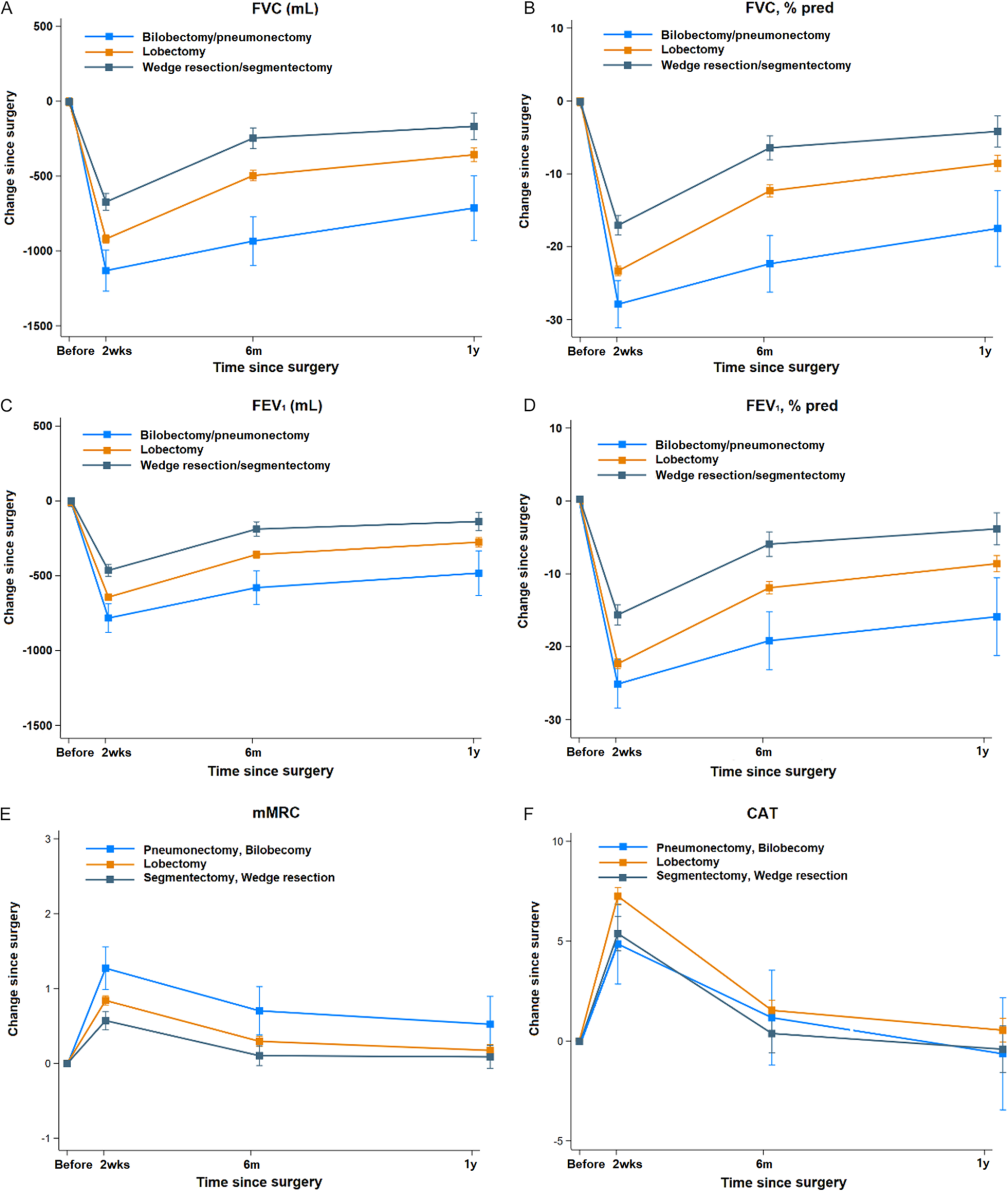
Change in pulmonary function and patient-reported outcomes by type of surgery and postoperative time. (**A**) FVC (mL), (**B**) FVC (percent of the predicted value), (**C**) FEV_1_(mL), (**D**) FEV_1_ (percent of the predicted value), (**E**) mMRC dyspnea scale, (F) CAT according to type of surgery and postoperative time *CAT* chronic obstructive pulmonary disease assessment test, *FEV*_*1*_ forced expiratory volume in 1 s; *FVC* forced expiratory vital capacity, *mMRC dyspnea scale* modified Medical Research Council dyspnea scale. *P for interaction (*P* < 0.01) between type of surgery (reference: lobectomy) and time after adjustment for age, sex, smoking status, obesity, stage, cell type, type of surgery, video-assisted thoracic surgery, postoperative pulmonary complications, and adjuvant treatment

The baseline levels of pulmonary function were similar, regardless of the type of surgery (Table 2). In patients with bilobectomy/pneumonectomy, FVC were decreased compared to those at baseline (-1195.6 mL, -999.6 mL, and -770.1 mL at 2 weeks, 6 months, and 1 year after surgery, respectively). Furthermore, patients with wedge resection/segmentectomy showed decreased FVC of -670.1 mL, -244.4 mL, and -164.7 mL at 2 weeks, 6 months, and 1 year after surgery, respectively (Table 2, Fig. 3). When we compared the changes from baseline of FVC (mL and % pred) by surgical extent, the changes from baseline of FVC (mL and % pred) in patients who underwent lobectomy were greater than those with wedge resection/segmentectomy, but were smaller than those with bilobectomy/pneumonectomy at 2 weeks, 6 months, and 1 year after surgery (Table 2). A similar pattern was observed for FEV_1_ (mL and % pred) and DL_CO_ (ml/min/mmHg and % pred). The prevalence of restrictive patterns at baseline was 4.9%, 6.8%, and 26.7% in the wedge resection/segmentectomy, lobectomy, and bilobectomy/pneumonectomy groups, respectively. During follow-up, the prevalence of restrictive patterns at 2 weeks, 6 months, and 1 year after surgery was higher than baseline, but the prevalence of obstructive patterns was similar in all surgery types (Table 3). In sensitivity analyses of participants with normal lung function at baseline, similar pattern was observed. Incidences of restrictive patterns in patients with lobectomy was lower than those with bilobectomy/pneumonectomy and was higher in patients with wedge resection/segmentectomy at 2 weeks, 6 months, and 1 year after surgery and (See Additional file 2).

Bilobectomy/pneumonectomy patients had the highest mMRC dyspnea grade among the three groups, but the difference was not statistically significant one year after surgery (Table 4). The odds ratio for a CAT score > 10 was significantly higher in patients with bilobectomy/pneumonectomy than those with lobectomy or wedge resection/segmentectomy at 2 weeks after surgery; however, this difference was not statistically significant at 6 months and 1 year after surgery (Additional file 3).

## Discussion

In this study, we investigated the changes in pulmonary function and PROs over one year after lung cancer surgery. We demonstrated that all parameters of lung function, dyspnea scale, and CAT scores noticeably worsened 2 weeks after surgery. During the follow-up period, lung function and PROs partly recovered but did not fully return to baseline. The pattern of alteration in PROs was found to be closely linked to changes in pulmonary function. Although the CAT scores fully recovered one year after surgery, the breathlessness and energy domains deteriorated. Lung function declines, along with worsening PROs including dyspnea, were more evident in patients who underwent bilobectomy or pneumonectomy. To our knowledge, this is the largest prospective cohort study with repeated (over 1 year) evaluations of pulmonary function and PROs after lung cancer surgery.

Surgical resection offers the best long-term survival results in patients with resectable NSCLC, but it also leads to the loss of lung parenchyma, with subsequent impairment of pulmonary function and worsening of PROs [7, 28–30]. Immediately after surgery, the FVC and FEV_1_ values dramatically decreased with worse PROs, including dyspnea. The proportion of patients with mMRC ≥ 2 was only 2.4% at baseline, which increased to 26.3% 2 weeks after surgery. CAT scores also showed a similar pattern, with a significant increase (more than double; 6–13) 2 weeks after surgery. Our results were in line with previous studies showing that patients experienced pain, fatigue, cough, and dyspnea during the first month after surgery [29–34]. The loss of pulmonary function was evidently correlated with the amount of lung resection; at postoperative 2 weeks, the decline in % pred of FVC and FEV_1_ was 17% and 15.5% after wedge resection/segmentectomy, 23.3% and 21.7% after lobectomy, and 28.8% and 24% after bilobectomy or pneumonectomy, respectively. Furthermore, approximately half of patients with bilobectomy/pneumonectomy had dyspnea with mMRC ≥ 2, and even after wedge resection/segmentectomy, more than 18% of patients suffered from dyspnea with mMRC ≥ 2 in the early postoperative period (Additional file 3). Our study indicated that patients undergo substantial symptomatic discomfort including dyspnea with a profound decrease of pulmonary function in the early postoperative period independent of the extent of surgery.

Six months after surgery, the decline in lung function and worsened PROs partially recovered, which is consistent with the results of previous studies [6–9, 28–30, 35, 36]. Owing to surgical incision and pain, pulmonary function could have been impaired more than expected in the early postoperative period. Thus, approximately 3–6 months would be required to overcome the postoperative residual pleural space (occupied by pleural fluid), achieve lung expansion, and displace the mediastinum and diaphragm [37]. Accordingly, symptoms were more relieved 6 months after surgery in our study, but the remaining significant symptoms varied according to the extent of surgery. While 8% of the patients showed significant dyspnea of mMRC ≥ 2 after lobectomy, 16% of the patients with bilobectomy/pneumonectomy still had dyspnea of mMRC ≥ 2 after surgery at 6 months. These results were similar to those of a previous study showing that patients who underwent pneumonectomy experienced greater dyspnea and pain than those who underwent lobectomy [32].

One year after surgery, lung function loss and worsening of symptoms were mitigated, regardless of the surgical extent. Regarding lung function, the remaining FVC (% pred) and FEV_1_ (% pred) losses one year after surgery were 4.1% and 4% after wedge resection/segmentectomy, 8.5% and 8.4% after lobectomy, and 18.2% and 14.4% after bilobectomy/pneumonectomy, respectively. However, 25% of patients with lobectomy and approximately half patients with bilobectomy/pneumonectomy still had restrictive patterns one year after surgery. In terms of PROs, approximately 5% of the patients had significant dyspnea with mMRC ≥ 2 1 year after surgery, and the proportion was not significantly associated with the extent of surgery. Other symptoms such as cough, sputum, chest tightness, confidence, and home activities were restored after 1 year compared to baseline, but the lack of energy and breathlessness persisted. Although lung function loss and dyspnea persisted in some patients 1 year after surgical resection, our results provide meaningful information on lung function and PRO changes over one year after surgery, based on surgical extent. Our results could guide patients on the timeline of improvement in lung function and PROs, especially from 2 weeks after surgery.

Postoperative impairment of pulmonary function has been reported to be an indicator of dyspnea [38]. The presence of dyspnea is associated with a low level of QOL, including physical, social, and role functions [12, 30]. The relationship between subjective symptoms and objective pulmonary function has been reported among patients with chronic lung disease [39–41], but it has rarely been reported in lung cancer patients postoperatively. In a previous study, respiratory symptoms were significantly more common in the presence of moderate-to-severe pulmonary dysfunction [38], but postoperative symptoms, such as dyspnea, are often ignored and underestimated. Despite these symptoms, medication usage was not commonly reported, and only 18% of the patients reported use of prescribed bronchodilators [38]. Several previous studies have shown that exercise programs are effective in improving exercise capacity, symptoms, and QOL after lung cancer surgery [42, 43]; however, the clinical application of exercise programs is still limited. To our knowledge, this is the largest prospective study to show the longitudinal changes in PROs related to postoperative lung function decline. Our data showed the nature of pulmonary function changes and symptoms over 1 year after lung cancer surgery, and found that the most deteriorated PROs and pulmonary function reductions occurred at 2 weeks, which could be targets for intervention. Deterioration of pulmonary function and related symptoms could be relieved or mitigated with integrated programs, including medication and rehabilitation. Future research is required to establish interventions to improve pulmonary function and patient discomfort.

The present study has several limitations. First, as the study was conducted only in patients at a tertiary hospital, the results might not represent different settings. Second, few patients dropped out during follow-up; hence, changes in pulmonary function could be overestimated if those patients showed worse performance. Third, the surgical procedures largely depended on the tumor characteristics, and differences in baseline characteristics according to surgical procedure were unavoidable. Finally, although the CAT is largely accepted among patients with chronic obstructive pulmonary disease, its clinical application in postoperative lung cancer patients has not been fully established. However, in this study, the Cronbach’s alpha of the CAT was 0.77, which is an acceptable value [44]. In terms of the mMRC dyspnea scale, which only involves one item, the correlation coefficient as convergence validity between CAT score and mMRC dyspnea scale was 0.49, which is also acceptable [45].

## Conclusions

In conclusion, our study demonstrated longitudinal changes in pulmonary function and integrated PROs in a large prospective cohort after lung cancer surgery. Lung function and PROs improved over time, but patients suffered from dyspnea and symptoms along with sharply decreased lung function in the early postoperative period, independent of the extent of surgery. Thus, physicians are required to stay attentive regarding lung function decline and associated symptoms after surgery and could provide proper information with emotional support as their lung function and QOL are expected to improve with time. Further studies are needed to establish intervention programs for these patients.

## Supplementary Information

**Additional file 1. **The formula of mixed effects model.

**Additional file 2: Table S1. **Changes in FEV_1_/FVC and patterns of ventilatory defect from baseline to 2 weeks, 6 months, and 1 year after surgery among patients with normal lung function before surgery (N = 431).

**Additional file 3: Table S2.** Changes in patient-reported outcomes from baseline to 2 weeks, 6 months, and 1 year according to the type of surgery.

## Data Availability

The datasets used and/or analyzed during the current study are available from the corresponding authors (Dr Hong Kwan Kim or Hye Yun Park) in response to reasonable requests.

## Abbreviations

NSCLC: Non-small cell lung cancer
FEV_1_: Forced expiratory volume in 1 s
QOL: Quality of life
HRQOL: Health-related quality of life
PROs: Patient-reported outcomes
DLco: Diffusing capacity of the lung for carbon monoxide
FVC: Forced vital capacity
mMRC: Modified Medical Research Council
CAT: Chronic Obstructive Lung Disease Assessment Test
% pred: Percent of the predicted values
