# Supplementary material for: Longitudinal changes in pulmonary function and patient-reported outcomes after lung cancer surgery
Source: Respir Res. 2022 Aug 30;23:224. doi: 10.1186/s12931-022-02149-9 (PMC9429784; doi:10.1186/s12931-022-02149-9)
Supplement: Supplementary file 1 — Additional file 1. The formula of mixed effects model. [file 12931_2022_2149_MOESM1_ESM.docx]

**Additional file 1**

**The formula of mixed effects model**

Where Time_ij_ denotes the timing, in years, of the measure of lung function on the i^th^ individual at the j measurement. Note that the expression for lobectomy closely resembles the equation for a linear mixed effects model presented earlier in the section, whereas the expression for current smokers contains 2 additional fixed effects, *β*_2_ and *β*_3_, which allow different population-averaged intercepts and slopes, respectively, for of wedge resection/segmentectomy and bilobectomy/pneumonectomy. In this model, *β*_1_ is the population mean change in lung function per visit for the lobectomy group; the corresponding rate of change in FEV_1_ per visit for the wedge resection/segmentectomy and bilobectomy/pneumonectomy group is *β*_1_+*β*_3_. Therefore, *β*3 is the population difference in the rates of change in mean lung function per year between the type of surgery groups. The question of main interest canters on the magnitude of *β*_3_. In this study, time was included in the model as categorical variable (baseline, 2 weeks, 6 months, and 1 year after surgery).
