# Supplementary material for: Longitudinal changes in pulmonary function and patient-reported outcomes after lung cancer surgery
Source: Respir Res. 2022 Aug 30;23:224. doi: 10.1186/s12931-022-02149-9 (PMC9429784; doi:10.1186/s12931-022-02149-9)
Supplement: Supplementary file 2 — Additional file 2: Table S1. Changes in FEV1/FVC and patterns of ventilatory defect from baseline to 2 weeks, 6 months, and 1 year after surgery among patients with normal lung function before surgery (N = 431). [file 12931_2022_2149_MOESM2_ESM.docx]

**Additional file 2**

Table S1 Changes in FEV_1_/FVC and patterns of ventilatory defect from baseline to 2 weeks, 6 months, and 1 year after surgery among patients with normal lung function before surgery (N = 431)

|  | |  | |  | 2 weeks after surgery | 6 months after surgery | 1 year after surgery |
| --- | --- | --- | --- | --- | --- | --- | --- |
| **Overall** | | | |  |  |  |  |
|  | | Obstructive pattern**, n (%)** | | | 44 (10.4) | 46 (12.0) | 46 (12.3) |
|  | | Restrictive pattern**, n (%)** | | | 261 (61.7) | 114 (29.8) | 76 (20.4) |
| **Type of surgery** | | | | |  |  |  |
|  | Obstructive pattern | | | |  |  |  |
|  |  | | Wedge resection/segmentectomy**, n (%)** | | 4 (4.7) | 5 (6.3) | 5 (6.3) |
|  |  | | Lobectomy**, n (%)** | | 38 (11.5) | 40 (13.6) | 40 (13.9) |
|  |  | | Bilobectomy/pneumonectomy**, n (%)** | | 2 (25.0) | 1 (14.3) | 1 (16.7) |
|  | *P*-value | | | | 0.05 | 0.15 | 0.12 |
|  | Restrictive pattern | | | |  |  |  |
|  |  | | Wedge resection/segmentectomy**, n (%)** | | 41 (48.2) | 10 (12.5) | 6 (7.6) |
|  |  | | Lobectomy**, n (%)** | | 214 (64.9) | 98 (33.2) | 66 (22.9) |
|  |  | | Bilobectomy/pneumonectomy**, n (%)** | | 6 (75.0) | 6 (85.7) | 4 (66.7) |
|  |  | | *P*-value | | 0.014 | < 0.01 | < 0.01 |
