# Supplementary material for: Longitudinal changes in pulmonary function and patient-reported outcomes after lung cancer surgery
Source: Respir Res. 2022 Aug 30;23:224. doi: 10.1186/s12931-022-02149-9 (PMC9429784; doi:10.1186/s12931-022-02149-9)
Supplement: Supplementary file 3 — Additional file 3: Table S2. Changes in patient-reported outcomes from baseline to 2 weeks, 6 months, and 1 year according to the type of surgery. [file 12931_2022_2149_MOESM3_ESM.docx]

**Additional file 3**

Supplementary Table 2. Changes in patient-reported outcomes from baseline to 2 weeks, 6 months, and 1 year according to the type of surgery

|  | |  | |  | Before surgery | 2 weeks after surgery | 6 months after surgery | 1 year after surgery |
| --- | --- | --- | --- | --- | --- | --- | --- | --- |
| Overall | | | | |  |  |  |  |
|  | | mMRC dyspnea grade ≥ 2 | | |  |  |  |  |
|  | |  | | Adjusted proportion (SE) | 2.4 (0.6) | 26.3 (1.8) | 7.9 (1.2) | 5.2 (1) |
|  | |  | | OR comparing baseline (95% CI)^a^ | Reference | 15.2 (8.5, 27.3) | 3.3 (1.8, 6.1) | 2.1 (1.1, 4.0) |
|  | | CAT ≥ 10 | | |  |  |  |  |
|  | |  | | Adjusted proportion (SE) | 21.6 (1.6) | 59.2 (2.0) | 20.1 (1.9) | 18.9 (1.8) |
|  | |  | | OR comparing baseline (95% CI)^a^ | Reference | 6.0 (4.7, 7.6) | 1.4 (1.4, 1.8) | 0.8 (0.6, 1.1) |
| Type of surgery | | | | |  |  |  |  |
|  | mMRC dyspnea grade ≥ 2 | | | |  |  |  |  |
|  | Adjusted proportion (SE) | | | |  |  |  |  |
|  |  | | Wedge resection/segmentectomy | | 1.7 (1.2) | 18.2 (3.7) | 5.5 (2.3) | 4.6 (2.2) |
|  |  | | Lobectomy | | 2.6 (0.7) | 27.4 (2.1) | 8 (1.4) | 5.3 (1.2) |
|  |  | | Bilobectomy/pneumonectomy | | 0 (0) | 45.6 (11.2) | 16.3 (8.5) | 5.6 (5.4) |
|  |  | | P values | | 0.85 | 0.03 | 0.31 | 0.97 |
|  | OR comparing baseline (95% CI)^a^ | | | |  |  |  |  |
|  |  | | Wedge resection/segmentectomy | | Reference | 13.5 (3.2, 57.8) | 3.4 (0.7, 15.3) | 3.0 (0.7, 13.4) |
|  |  | | Lobectomy | | Reference | 15.3 (8.5, 27.5) | 3.2 (1.8, 5.8) | 1.9 (1.0, 3.7) |
|  |  | | Bilobectomy/pneumonectomy | | Reference | 47.7 (3.4, 661.1) | 8.4 (0.6, 116.1) | 3.0 (0.7, 13.4) |
|  |  | | P for interaction | |  | 0.82 | 0.89 | 0.59 |
|  | CAT ≥ 10 | | | |  |  |  |  |
|  | Adjusted proportion (SE) | | | |  |  |  |  |
|  |  | | Wedge resection/segmentectomy | | 24.3 (3.9) | 50.9 (4.6) | 19.3 (3.9) | 16.5 (3.8) |
|  |  | | Lobectomy | | 20.1 (1.8) | 61.5 (2.3) | 29.1 (2.2) | 19.2 (2.0) |
|  |  | | Bilobectomy/pneumonectomy | | 39.3 (10.1) | 57.3 (10.7) | 52.8 (11.5) | 26.9 (10.9) |
|  |  | | P values | | 0.07 | 0.12 | 0.01 | 0.60 |
|  | OR comparing baseline (95% CI)^a^ | | | |  |  |  |  |
|  |  | | Wedge resection/segmentectomy | | Reference | 13.4 (3.1, 57.4) | 3.4 (0.7, 15.3) | 2.8 (0.6, 12.8) |
|  |  | | Lobectomy | | Reference | 15.2 (8.5, 27.3) | 3.3 (1.8, 6.1) | 2.1 (1.1, 4.0) |
|  |  | | Bilobectomy/pneumonectomy | | Reference | 33.7 (3.5, 32.3) | 7.1 (0.8, 63.1) | 2.1 (1.1, 4.0) |
|  |  | | P for interaction | |  | <0.01 | 0.03 | 0.37 |

Baseline values were used as reference.

*Adjusted for age, sex, stage, obesity, smoking status, cell type, type of surgery, video-assisted thoracic surgery, postoperative pulmonary complications, and adjuvant treatment.

CAT, chronic obstructive pulmonary disease assessment test; CI, confidence interval mMRC dyspnea scale, modified Medical Research Council dyspnea scale; SE, standard error; OR, odds ratio.
